# Efficacy of piezoelectric surgery in the conservative management of unicystic ameloblastoma: a 5-year retrospective cohort study

**DOI:** 10.1186/s12903-026-07760-6

**Published:** 2026-01-26

**Authors:** Alessandro Antonelli, Selene Barone, Elena Calabria, Vincenzo Greco, Antonio Madonna, Francesco Bennardo, Giulia Brunello, Amerigo Giudice

**Affiliations:** 1 School of Dentistry, Department of Health Science, Magna Graecia University of Catanzaro, Viale Europa, 88100 Catanzaro, Italy; 2 Department of Oral Surgery, University Hospital of Düsseldorf, Düsseldorf, Germany; 3 Department of Orthodontics and Dentofacial Orthopaedics, Charité - Universitätsmedizin Berlin, corporate member of Freie Universität Berlin and Humboldt-Universität zu Berlin, Berlin, Germany

**Keywords:** Ameloblastoma, Odontogenic tumors, Piezosurgery, Conservative treatment, Recurrence

## Abstract

**Background:**

Unicystic ameloblastoma (UAM) is a subtype of ameloblastoma characterized by less aggressive clinical behavior than its conventional counterpart. Surgical enucleation represents a conservative approach compared to more invasive treatment options; however, the risk of recurrence may be significant. The aim of this study was to evaluate the efficacy of the conservative treatment of UAM associated to a piezoelectric debridement. The main outcome was to evaluate the recurrence rate of UAM over a 5-year follow-up period.

**Methods:**

A retrospective cohort study was conducted on patients diagnosed with UAM who underwent enucleation and residual cavity debridement with a piezoelectric surgery device. Clinical and radiological follow-ups were conducted at 6-month intervals over a 5-year period.

**Results:**

Twelve patients (7 female and 5 men) met the inclusion criteria and completed the 5-year follow-up. Lesions predominantly affected the mandible (10 cases), with the posterior region being the most common site. Recurrence occurred in 2 out of 12 patients and was limited to the mural variant of UAM. Both recurrent cases occurred in the posterior mandible and involved the largest lesion sizes observed in this study.

**Conclusions:**

The conservative approach of UAM combining enucleation and piezoelectric bone debridement provided a minimally invasive and effective solution for the management of this pathology, representing a viable alternative to surgical resection.

## Introduction

Unicystic ameloblastoma (UAM) is one of the most common subtypes of ameloblastoma, second only to the conventional type, and occurs in patients with a mean age of 26.1 years [1]. This tumor usually manifests as an intraosseous lesion with an epithelial lining resembling that of conventional ameloblastoma [2]. The relative frequency of UAM ranges from 5% to 22% of all ameloblastoma cases, although some authors reported a higher frequency of 31.1% [3].

In 1988 Ackermann et al. [4] identified three variants of UAM: luminal, intraluminal, and mural. The luminal variant is characterized by a cystic lesion lined with ameloblastic epithelium. In the intraluminal type, the cyst lining exhibits polypoid proliferation projecting into the lesion lumen. The mural type involves infiltration of the fibrous wall of the cyst by ameloblastic epithelium and is associated with recurrence rate similar to that of the more aggressive conventional ameloblastoma [5–8].

Biopsy of the lesion plays a main role in the differential diagnosis of UAM, as many other lesions share similar clinical features [7], such as dentigerous cysts, odontogenic keratocysts, ameloblastic fibromas, adenomatoid odontogenic tumors, and odontogenic fibromas [9].

In the context of ameloblastoma treatment, therapeutic management remains controversial. For this reason, various strategies have been explored to address different subtypes, each balancing recurrence risks and postoperative outcomes [10]. For UAM, conservative therapy such as enucleation is commonly employed; however, this treatment approach could be associated with a higher recurrence rate [11]. Conversely, more radical surgery, such as resection, reported a lower recurrence rate but is linked to a greater incidence of postoperative complications [11]. This surgical approach often leads to greater patient disability, significantly impacting their quality of life by adversely affecting speech, chewing ability, and social interactions [12]. Moreover, in a recent systematic review it was found only a weak correlation between resection and a lower recurrence rate in the treatment of UAM, suggesting that treatment choice should be carefully considered given the radical surgery-associated complications [13]. Specifically, recurrence rates were reported as 3.6% for radical approaches, 30.5% for enucleation, 18% for marsupialization, and 16% for enucleation followed by the application of Carnoy’s solution [13]. The variability in recurrence rates suggests a potential relationship with the histopathologic variant of UAM. Notably, the luminal and intraluminal variants exhibit lower recurrence rates following conservative treatment compared to the mural variant [14].

Moreover, an increased recurrence rate of UAM has been related to clinicopathological indicators of local aggressiveness, such as root resorption and invasion of the bone cortex and adjacent soft tissues. The higher risk of recurrence may be attributable to the persistence of residual epithelial nests beyond the cystic lining following conservative treatment, highlighting the rationale for careful debridement of residual tumor cells, particularly in the presence of these aggressive features [15].

To date, various approaches have been described in the conservative management of UAM, including enucleation, marsupialization, enucleation with the application of Carnoy’s solution, and enucleation combined with bone curettage [13]. In the latter approach, which is based on the removal of the UAM lesion and any residual epithelial cells in the bone cavity walls, many authors suggested performing the bone debridement using piezoelectric devices [16]. This surgical approach was specifically developed for bone surgery to enable a less invasive procedure, promoting better postoperative recovery while ensuring precise and selective bone cutting with minimal trauma [17, 18]. Moreover, several studies have shown the effectiveness of the piezoelectric osteotomy in minimizing damages to surrounding tissues and reducing the incidence of necrosis [18].

Several authors have reported the advantages of piezoelectric surgery in the management of odontogenic lesions, emphasizing its superior safety profile in critical areas, such as in proximity to the maxillary sinus and mental nerve [18, 19]. Additionally, this technique is associated with reduced intraoperative bleeding, a lower risk of lesion perforation during enucleation and lower recurrence rates [19].

Currently, there is no clear consensus in the literature on the more predictable protocol for removing residual pathological cells after the enucleation of UAM [13].

Although some studies have reported the treatment of UAM through enucleation and curettage of the peripheral bone margins, they do not provide specific details on whether this procedure was performed manually, with rotary instruments, or using piezoelectric surgery [8, 20]. For this reason, a retrospective clinical study was designed to evaluate the efficacy of conservative treatment of UAM using piezoelectric surgery.

The study aimed to assess the long-term outcomes and recurrence rates over a 5-year follow-up period.

## Materials and methods

### Study design

The study was designed as a retrospective single-center cohort study and it was conducted in accordance with the Declaration of Helsinki and approved by the Regional ethical review board (reference for Magna Graecia University of Catanzaro, Catanzaro, Italy - n. 301/2024). 

### Study sample and preoperative procedure

The medical records of patients diagnosed and treated for UAM at the Oral Surgery and Pathology Unit of ‘Renato Dulbecco’ Hospital, Magna Graecia University of Catanzaro, between January 2014 and April 2020, were retrospectively analyzed. The inclusion criteria of the study were as follows: (i) patients who underwent conservative treatment for UAM, including lesion enucleation and piezoelectric surgical debridement; (ii) availability of radiological assessments; (iii) availability of microscopic findings for all excised specimens; (iv) a minimum follow-up duration of 5 years. The exclusion criteria were as follows: (i) patients diagnosed with UAM who were treated with alternative conservative approaches (enucleation alone, marsupialization, enucleation with the application of Carnoy’s solution, enucleation combined with bone curettage performed manually or with rotary instruments) or resective surgery; (ii) incomplete medical or radiological records; (iii) incomplete follow-up data.

A comprehensive clinical history was collected from the files available in the medical record section for each patient. Relevant demographic information (age, sex) and any present symptoms were noted. Furthermore, for each patient the following parameters were recorded: the anatomical location of the lesion, the maximum lesion size, the presence of bone cortical plate expansion, the presence of any impacted teeth and the presence of neurological alterations at the time of surgery. The maximum lesion size referred to the largest dimension observed on preoperative Cone Beam Computed Tomography (CBCT), identified among the anteroposterior, mediolateral, and craniocaudal measurements.

At the time of the initial consultation, patients presented with orthopantomography (OPT) or intraoral radiographs, which served as the first-line imaging modality. These radiographs revealed unilocular radiolucency and provided preliminary information regarding the lesion and any associated impacted teeth. Subsequently, preoperative radiological assessment included CBCT, which allowed for a detailed evaluation of the lesion’s extent and its relationship with adjacent anatomical structures, thereby enabling more accurate radiological assessment and facilitating precise surgical planning. For each lesion a preoperative incisional biopsy was performed to confirm the diagnosis of the lesion.

### Surgical procedures and follow-up

All patients enrolled in this study had a histological assessment of UAM according to the criteria defined by the World Health Organization (WHO) classification [6].

**Fig. 1:**
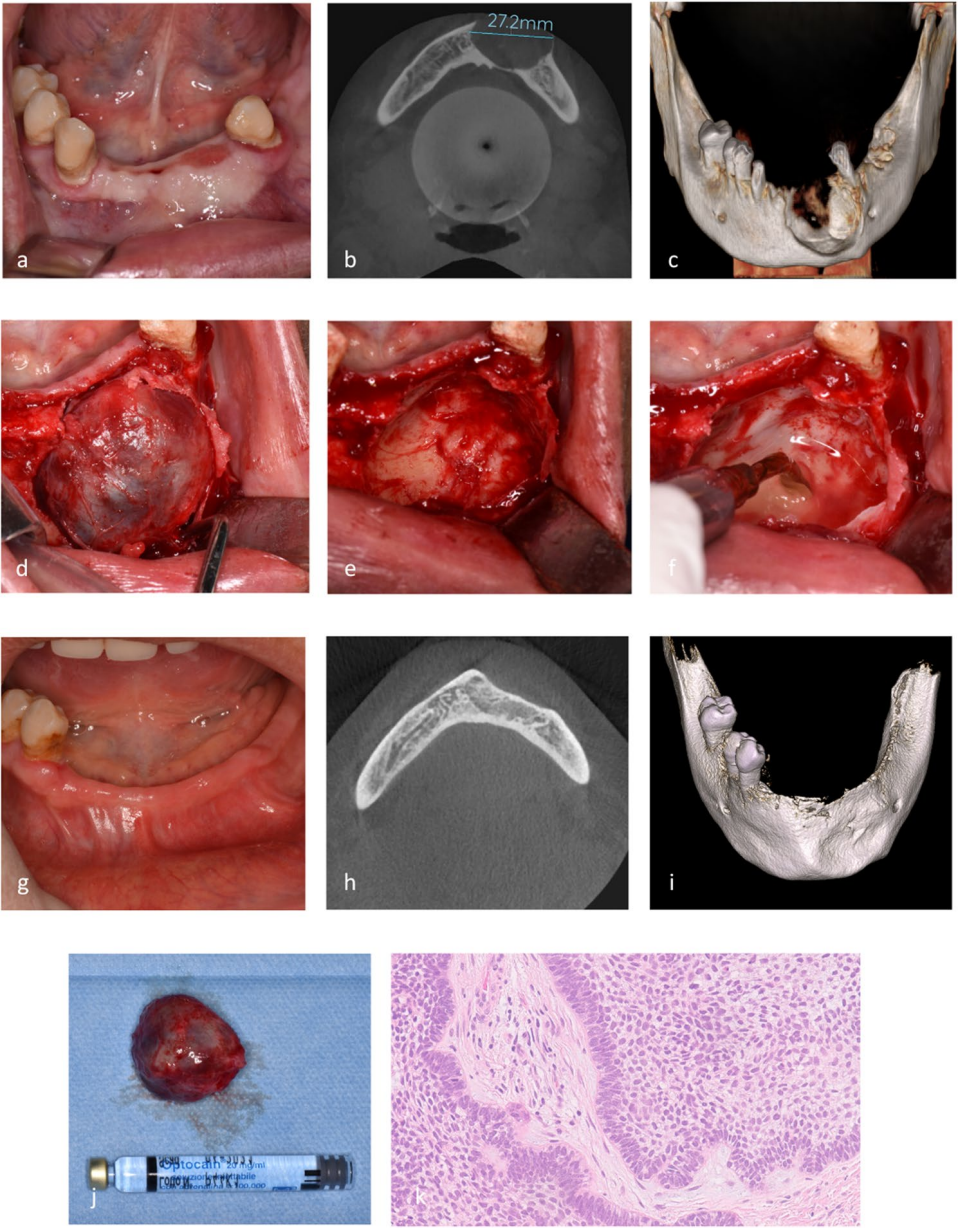
Conservative treatment of Unicystic Ameloblastoma (UAM) - Case 1. (**a**): Preoperative intraoral clinical image showing swelling in the mandible; (**b**): Preoperative axial CBCT scan showing a well-defined radiolucent lesion; (**c**): Preoperative 3D CBCT reconstruction showing the expansion of the cortical plate; (**d**): Intraoperative view after mucoperiosteal flap elevation and enucleation of the lesion; (**e**): Residual cavity of the lesion after enucleation, revealing epithelial debris and crypts; (**f**): Piezosurgical debridement of the peripheral bone; (**g**): 5-year clinical follow-up; (**h**): 5-year CBCT follow-up exhibiting bone healing and no recurrence; (**i**): 5-year 3D CBCT reconstruction showing mandibular bone tissue healing; (**j**): Enucleated lesion sample preserved for histopathologic analysis; (**k**): Histological examination of the sample; a well-defined cystic lesion lined by basaloid to columnar ameloblast-like cells with peripheral palisading nucleus exhibiting focal reverse polarity. The neoplastic islands are detached from the luminal surface and embedded directly within the connective tissue (mural subtype)

**Fig. 2:**
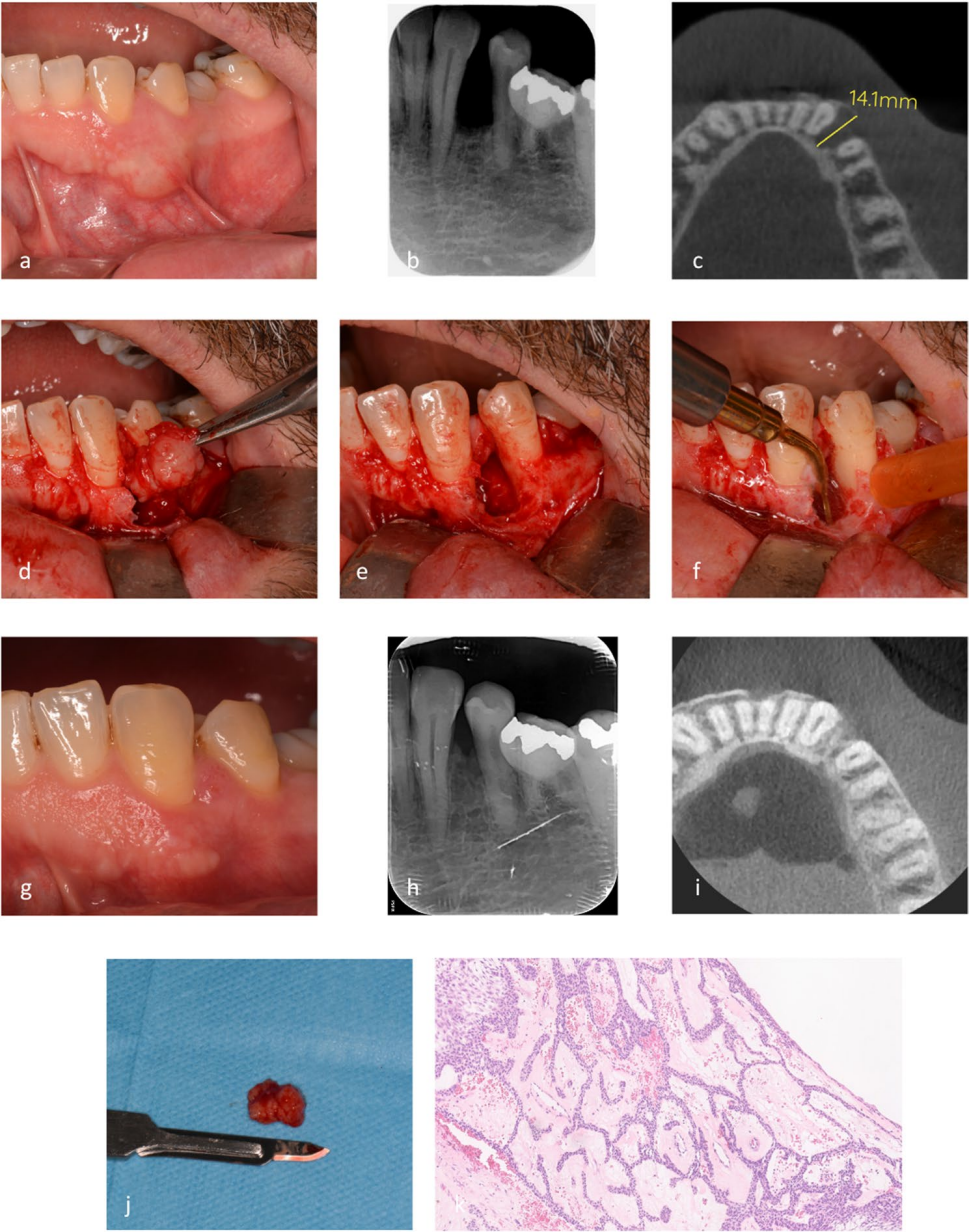
Conservative treatment of Unicystic Ameloblastoma (UAM) - Case 2. (**a**): Preoperative intraoral clinical image showing gingival swelling; (**b**): Preoperative intraoral X-ray showing a radiolucent lesion; (**c**): Preoperative axial CBCT scan revealing a well-defined lesion; (**d**): Intraoperative view after enucleation of the lesion; (**e**): Residual cavity after lesion enucleation (**f**): Piezosurgical debridement of the peripheral bone; (**g**): 5-year clinical follow-up after surgery; (**h**): Intraoral X-ray at 5-year follow-up evidencing bone healing; (**i**): 5-year CBCT follow-up showing absence of lesion recurrence; (**j**): Enucleated lesion sample preserved for histopathologic analysis; (**k**): Histological examination showing a cystic cavity lined by ameloblastoma-type epithelium, composed of a single or multilayered columnar basal cell layer, with an unremarkable stroma (luminal subtype)

Each surgical procedure was performed by a single experienced surgeon (A.G.) under local anesthesia. The same treatment planning and surgical protocol was executed for all UAM lesions. A mucoperiosteal flap was raised, followed by a perilesional osteotomy using piezoelectric device (PIEZOSURGERY^®^ white, Mectron S.p.A., Carasco, Italy) with dedicated insert (OP3 - Mectron S.p.A., Carasco, Italy) in areas where cortical bone was present. Each lesion was enucleated and dissected from the surrounding tissues using both manual instruments and piezoelectric devices. If impacted dental elements were present, they were extracted during the procedure. In all cases, the residual bone cavity was carefully debrided using the OP3 piezoelectric insert, both to remove the superficial peripheral bone layer and any residual epithelial tissue within the cavity (Figs. 1 and 2). No bone grafting was performed in any of the cases.

The collected specimen was preserved in a bottle containing 10% neutral buffered formalin and subsequently analyzed for definitive histopathologic diagnosis (Figs. 1 and 2).

The patients were examined at 6-month intervals over a period of 5 years. Routine radiographic follow-up was performed through CBCT evaluation one year after surgery, complemented by intraoral clinical examination via OPT or intraoral radiography every six months to detect any potential recurrence (Figs. 1 and 2).

### Statistical analysis

All data were collected using Microsoft Excel (Version 16.0, Microsoft Corporation, 2023). Statistical analysis was performed using R Studio software (250 Northern Ave, Suite 420, Boston, MA 02210) [21]. Descriptive statistics reported the mean and standard deviation for continuous quantitative variables, and absolute frequencies and percentages for categorical variables. A time-to-event analysis was conducted to assess recurrence-free rate after conservative treatment, which included enucleation and bone debridement with piezosurgery. Recurrence was defined as the event in focus, and the time interval was quantified in months. A Kaplan-Meier curve was created to descriptively show recurrence-free rate over the follow-up duration.

## Results

Twelve patients (5 males and 7 females), aged 18 to 40 years (mean age 27.1 ± 6.1 years) and diagnosed with UAM, were included in this study (Table 1). The cohort consisted of consecutive patients who met the inclusion criteria during the study period.

**Table 1:** Descriptive statistics of the study sample

Demographic Variables	Total number	Number with Recurrence
Age (years)		
<20	1	0
20–30	7	1
30–40	4	1
Gender		
Male	5	2
Female	7	0
Signs and Symptoms		
Swelling	7	2
Pain	5	1
Neurological alterations	1	0
Cortical plate expansion	7	2
Site		
Anterior Maxilla	0	0
Posterior Maxilla	2	0
Anterior Mandible	2	0
Posterior Mandible	8	2
Size		
<20 mm	3	0
20–30 mm	3	0
>30 mm	6	2
Impacted tooth		
Yes	3	1
No	9	1
Variant		
Luminal	4	0
Intraluminal	3	0
Mural	5	2

The average age of the patients was 28.2 ± 6.1 years, with a predominance of both female and male patients in the second decade of life.

The lesions were mainly located in the mandible (10 cases), with a 4:1 ratio between the posterior and the anterior region. UAM was identified in maxilla in only two cases, both located in the posterior region. Preoperative evaluation of the CBCT scans showed that the maximum lesion size ranged from 14.1 mm to 44.9 mm, with a mean measurement of 30 ± 9.3 mm.

Based on the definitive histology of the excised specimens, all the three histological variants of UAM were detected, with a predominance of the mural variant, observed in 5 out of 12 cases. The luminal variant was detected in 4 out of 12 cases, while the intraluminal variant was found in 3 out of 12 cases. Following excision, histological analysis corroborated the same pattern observed in the prior incisional biopsy in all cases.

**Fig. 3:**
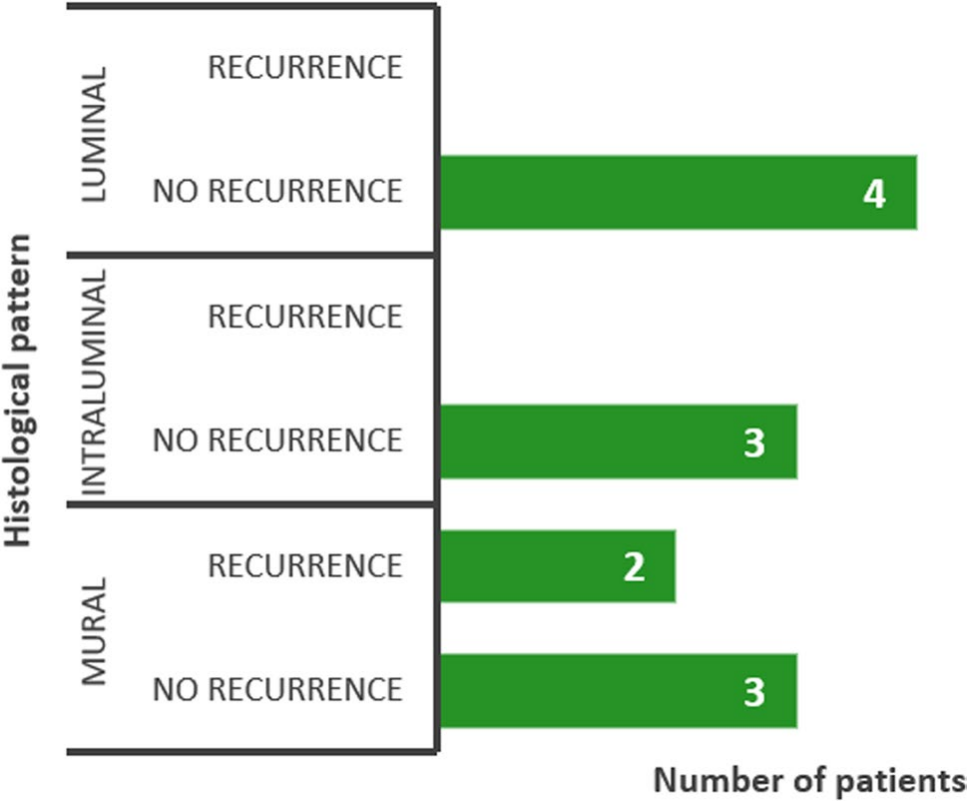
Five-year recurrence rates according to histological variants of UAM

Recurrence occurred in 2 out of 12 subjects. In both recurrent cases, the lesion was located in the posterior mandible and exhibited the mural histological pattern (Fig. 3).

**Fig. 4:**
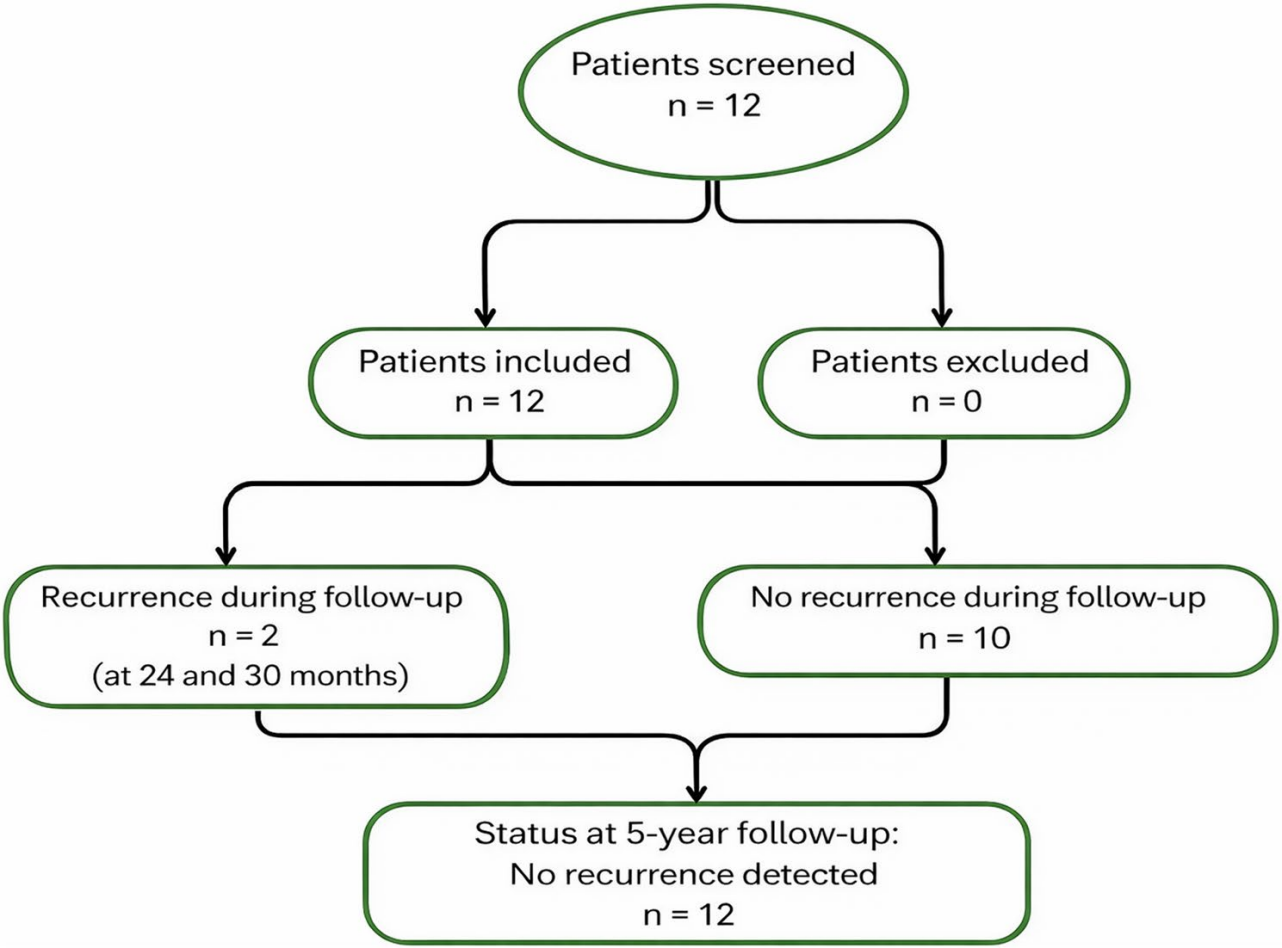
Flow diagram of included patients in the study, recurrence events, and long-term outcomes

The flow diagram presenting the included patients and recurrence cases is shown in Fig. 4.

Both recurrence cases were identified during routine clinical and radiological follow-up visits at 6-month intervals, and histological examination of the excised specimens confirmed the UAM diagnosis. In the two cases of recurrence, the histological variant was consistent with that of the initial lesion. One recurrence occurred during the 24-month follow-up, while the other was observed 30 months after this surgical approach. Following clinical and radiological identification, the recurrent lesions were surgically excised using the same conservative treatment. No further recurrences have been detected to date during a follow-up period of 5 years.

**Fig. 5:**
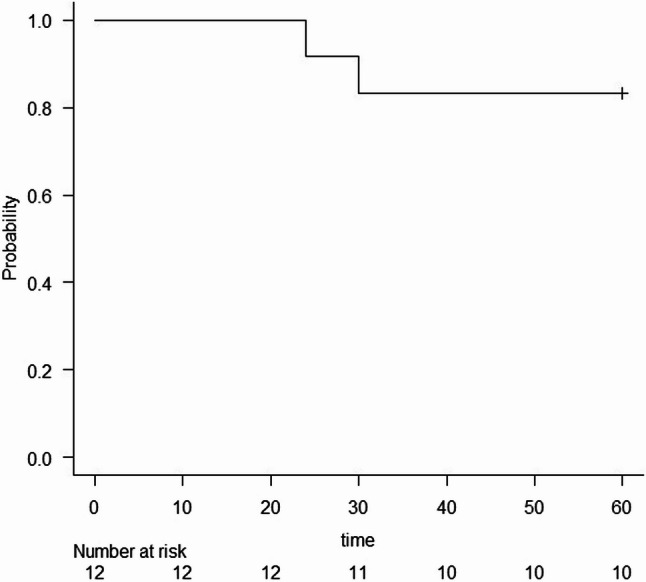
Kaplan–Meier curve illustrating recurrence-free rate after conservative treatment over a 5-year follow-up period, with an estimated recurrence-free probability of approximately 80% at the end of follow-up

The Kaplan–Meier curve indicated a recurrence-free rate of approximately 90% at 24 months and 80% at 30 months (Fig. 5). At the end of the 5-year follow-up, the probability of remaining recurrence-free persisted at nearly 80%. Given the limited sample size and the small number of events, the analysis was performed solely for descriptive use.

At the time of the initial consultation, the most common signs and symptoms reported by patients were swelling (7 cases) and pain (5 cases) (Table 1). Only one patient experienced temporary neurological alterations of the inferior alveolar nerve that resolved within four months after surgery (Table 1). Expansion of the cortical plate was observed in seven UAMs, five located in the mandible (two in the anterior region and three in the posterior region) and two in the maxilla (Table 1). The lesions were associated with three impacted teeth, i.e., two mandibular third molars and one mandibular canine (Table 1).

## Discussion

A careful assessment of the lesion signs is crucial for an accurate diagnosis and appropriate treatment of the UAM [22]. Radiological signs, such as root resorption of adjacent teeth, may suggest specific pathologies, but further examinations, including a biopsy, are required for a definitive diagnosis. In particular, a sample collection that includes both the lesion and surrounding tissue is mandatory, as UAM’s epithelial lining closely resembles that of dentigerous cysts, potentially leading to improper results if the biopsy procedure is not performed correctly [9]. Given the reliability of incisional biopsy as a diagnostic technique for intraosseous lesions, early biopsy evaluation of an adequate tissue sample, combined with clinical and radiological assessment, is essential to ensure optimal treatment planning [23]. The differential diagnosis of UAM includes other odontogenic cysts, such as follicular cyst, periodontal cysts and odontogenic keratocysts [7]. Once an accurate diagnosis is established, the primary concern is to reduce the recurrence rate of this pathology [24].

The present study aimed to evaluate the long-term effectiveness of a conservative treatment protocol for UAM, which involved piezoelectric surgical debridement following lesion enucleation. The efficacy of this protocol was assessed by analyzing the recurrence rate over a 5-year follow-up period.

Determining the most suitable surgical strategy for treating ameloblastoma remains a debated issue in literature [19, 25]. Many studies on different types of ameloblastoma favor more radical surgical procedures, such as marginal or segmental bone resections, due to the elevated recurrence rates seen in patients undergoing conservative management [26, 27]. However, it is essential to assess the potential risks associated with radical bone surgery, including the need for general anesthesia and its impact on both aesthetic and functional outcomes. The selection of the ideal treatment approach for managing ameloblastoma often requires consideration of the lesion’s nature and its histological variant [1, 28].

In the management of multicystic ameloblastoma, the preferred surgical approach typically involves wide segmental resection of the affected area with adequate safety margins to ensure complete removal of the lesion, often sacrificing critical anatomical structures such as nerves and vessels [29]. However, in cases of well-localized UAM, a more conservative surgical strategy can serve as a highly effective alternative [20]. This less invasive approach may help minimize postoperative discomfort and could offer significant advantages in preserving the patient’s functional and aesthetic outcomes over the long term [20, 30].

While several studies emphasize how the histological nature of the lesion plays a crucial role in influencing the likelihood of recurrence, an equally key factor is the surgical technique employed [31–33]. Specifically, the surgical ability in the removal any residual tissue during the procedures, following the enucleation of the lesion, is critical. This approach may reduce the risk of recurrence while ensuring optimal long-term outcomes in patient care [34].

In our study, recurrence was observed in 2 out of 12 cases over a five-year follow-up period.

This duration of follow-up aligns with the average timeframe reported in similar studies in the literature [13]. Notably, as highlighted by Reichart et al. [35], only 53% of recurrences are detected within the first five years after treatment, indicating that nearly half of all recurrences may occur beyond this period. Consequently, it is strongly recommended to extend patient follow-up well beyond the initial five years, ideally adopting a long-term or even lifelong monitoring approach to ensure timely detection and management of potential recurrences [35].

In a systematic review by Lau and Samman [13], various conservative surgical approaches for treating UAM were analyzed, revealing different recurrence rate. The authors found that enucleation alone resulted in a recurrence rate of 30.5%. In contrast, when enucleation was followed by the application of Carnoy’s solution, the recurrence rate dropped to 16%. Furthermore, marsupialization, with or without additional treatments in a second phase, was associated with a recurrence rate of 18% [13]. These findings highlight the varying effectiveness of different conservative treatments in managing UAM and preventing recurrence.

The application of Carnoy’s solution, penetrates the medullary space and provides an effect similar to surgical bed debridement with piezosurgery, targeting the residual tumor cells that have invaded the surrounding tissues [13, 36]. In fact, as stated by Marx et al. [37], ameloblastoma can extend between 2.3 and 8 mm beyond the radiographic boundary of the tumor. In particular, as reported by Ringer et Kolokythas [38], UAM showed medullary invasion tendency of approximately 0.25 cm from the tumor edge, with absence of pathological entities at a distance of 0.5 cm. Therefore, even if the tumor is completely enucleated, ameloblastic cells may persist in the surrounding tissues following lesion removal; therefore, adjunctive treatment of these areas can help reduce the risk of recurrence [39–41]. The invasive nature of this type of lesion is supported by Titinchi and Brennan [11], who, in a retrospective analysis of UAM management between 1995 and 2020, suggested that adding treatment to the peripheral bone margins after enucleation of the lesion could reduce the risk of medium- to long-term recurrence.

Another key factor that may contribute to the recurrence of UAM is represented by the histological subtype of the lesion. As highlighted by Ackermann et al. [4], lesions of the mural subtype of UAM exhibit a greater tendency to invade the surrounding bony trabeculae. This invasive behavior is associated with a markedly higher recurrence rate compared to other UAM variants, underscoring the relevance of surgical debridement of the surrounding areas in such cases.

Moreover, the tendency of the mural histologic variant towards recurrence is supported by Titinchi and Brennan [11], which found that four out of five recurrences following conservative treatments (enucleation alone or combined with Carnoy’s solution) were associated with the mural variant. This finding aligns with the results of our study, where both cases of recurrence (2 out of 12) involved the mural histologic variant.

In addition to the histologic variant, tumor size appears to be another relevant risk factor for recurrence [42]. According to Yang et al. [43], lesions larger than 60 mm are associated with an increased risk of recurrence. This finding is corroborated by the present study, which revealed that the two cases of mural variant recurrence exhibited anteroposterior, mediolateral, and craniocaudal lengths of approximately 60 mm. The remaining three cases out of five of the mural variants did not recur, likely due to their smaller size. On the other hand, the absence of recurrence in lesions with larger tumor sizes may be attributed to the intraluminal and luminal histologic variants.

Although many authors described various approaches for debriding the tissues surrounding the lesion in the conservative treatment of UAM, our study focused on the use of piezoelectric surgery following the enucleation of the lesion to minimize the recurrence risk [10, 44].

As widely documented in the existing literature, the piezoelectric surgery showed high effectiveness in the context of bone surgery [45–47]. This surgical device seems to enhance bone healing while preserving the integrity of the surrounding structures, and available immunohistochemical evidence suggests that it promotes greater osteoblastic viability compared with conventional bone surgery approaches [48–57]. Furthermore, piezosurgery exhibits microvibrational osteotomy properties that promote osteogenesis and postoperative bone regeneration while minimizing thermal damage to bone and neighboring structures, such as teeth. These microvibrations also produce reduced noise compared with the macrovibrations generated by rotary instruments, helping to decrease patient fear and stress during osteotomy procedures. The cavitation effect created by the microvibrations and the distribution of the cooling fluid effectively clears blood, improving visibility in the surgical field [58]. Additionally, the specific design of the insert used in this study (OP3 – Mectron S.p.A., Carasco, Italy) provided greater intraoperative control, enabling a targeted and minimally invasive osteoplasty. This contrasts with other inserts commonly used in oral surgery, which are primarily designed for more invasive procedures such as resective or segmental osteotomies. Troiano et al. conducted a comparative study on peripheral osteotomy techniques following enucleation of solid or multicystic ameloblastoma, evaluating the use of piezoelectric surgery versus conventional rotary instruments [44]. Notably, they observed a lower recurrence rate of the lesion in patients treated with piezoelectric surgery compared to those treated with rotary instruments. This finding indicates that piezoelectric approach may be more effective in reducing the risk of recurrence of these lesions [44]. In 2007, Covani et al. described a clinical case of a patient with an intraluminal unicystic ameloblastoma [16]. This lesion was treated conservatively with enucleation followed by rotary using a piezoelectric instrument, and no recurrence was observed at the 5-year follow-up [16]. Although it is a single case, this report is in line with the present study, as the intraluminal histologic variant did not recur in our research during the same follow-up period. This may be attributed to the use of the same surgical procedure employed in both studies [16]. Nevertheless, these comparisons of recurrence rates should be considered preliminary, as they are based on studies with small sample sizes.

In literature, the mechanisms involved in this phenomenon have been poorly investigated. According to recent findings, tumor cells may be susceptible to mechanical and oxidative damage as a result of cavitation, sonoluminescence, and piezoelectric-related catalytic processes [59]. This could potentially contribute to ultrasound-associated cytolytic activity mediated by reactive oxygen species (ROS). Recent research in piezoelectric technology have supported this hypothesis by showing how ultrasonic activation of piezoelectric materials can produce energy band modulation, which in turn might promote the synthesis of ROS at the cellular level. Through a combination of piezoelectric, sonodynamic, and catalytic effects, some studies have suggested potential improvements in cavitation strength, local piezoelectric potential, and ROS generation [59]. The biological plausibility that ultrasound-activated piezoelectric effects could contribute to tumor cell destruction and cytolysis is supported by preliminary preclinical in vitro and in vivo investigations showing tumor inhibition linked to these mechanisms [59, 60]. These results offer mechanistic insight into how ultrasound-driven piezoelectric events could contribute to tumor cells cytotoxicity through ROS-mediated pathways [60]. Although existing literature proposes these findings as a reasonable hypothesis for the impact of piezoelectric processes on tumor cells, the fundamental principles remain uncertain with limited supporting evidence. The results of our study suggest a potential role of the piezoelectric system in decreasing recurrence, not solely via ROS-induced apoptosis of tumor cells but also through its capacity to selectively clear the bone layer, which may contain residual epithelial islands penetrating the cancellous bone.

The relevance of removing lesion debris by scraping the bone walls after UAM enucleation is highlighted in a more recent case report, which underscores the importance of performing this procedure as a secondary surgical step following marsupialization [8]. Notably, the authors performed a third procedure specifically to remove necrotic bone and inflammatory tissue, emphasizing the need for meticulous removal of pathological tissue. Although it was not specified the debridement technique used, the absence of recurrence of the mural variant suggests that the scraping and the removal of all residual pathologic tissue are significant to minimize the recurrence risk [8].

In our study, we suggested that the piezoelectric surgical approach may be effective in the conservative treatment for UAM, with a low recurrence rate limited to the mural variant. Our results highlighted the benefit of less invasive strategy in treating UAM while minimizing the risk of recurrence. The use of a piezoelectric surgery device for the local debridement and removal of residual pathological tissue enhances surgical precision and may improve bone healing, thus reducing complications occurrence. Furthermore, considering the recent evidence highlighting the potential cytotoxicity associated with the use of Carnoy’s solution following the enucleation of odontogenic lesions and the comparable action on remaining tumor cells, the use of piezoelectric surgery may offer an additional benefit in the debridement of residual cavities after UAM removal, minimizing the risk of tissue toxicity [61].

### Study limitations

The small cohort of 12 patients represents a limit of our research and a larger sample size may provide more consistent results. Furthermore, although the follow-up period aligns with that reported in similar studies, it may not be sufficient to detect late recurrences occurring beyond five years. This limitation underscores the relevance of prolonged patient monitoring to ensure the timely identification and management of late recurrences following conservative treatment of UAM. Another limitation of the present study is the absence of a control group, and prospective multicenter studies should consider comparing outcomes between enucleation with piezoelectric bone curettage and other alternative treatments.

Moreover, specific in vitro and in vivo studies are needed to evaluate the direct effects of piezoelectric surgery on UAM cells and surrounding bone structures.

Future large-scale studies should also aim to evaluate the individual and combined effects of lesion dimension, histological subtype, and location on the recurrence rate of UAMs when a conservative approach using piezoelectric surgery is adopted.

## Conclusion

The conservative management of UAM, which involves enucleation followed by piezoelectric curettage of the residual bone walls and regular follow-up, may represent a feasible alternative to more invasive surgical resection, particularly in cases involving non-mural histological subtypes and lesions with reduced extension. The piezoelectric device is particularly advantageous in this approach, as it can enable precise and mini-invasive debridement of the bony surfaces surrounding the lesion. By facilitating the removal of epithelial debris and the achievement of smoother bone walls, this approach could potentially contribute to reducing the risk of recurrence of this pathological lesion. Furthermore, this technique supports a low long-term recurrence rate while minimizing short-term postoperative complications, presenting it as a viable and minimally invasive surgical approach. Based on our data, UAM may be treated with a less-radical approach that combines enucleation and piezoelectric bone curettage. However, lesions larger than 30 mm may carry a higher risk of recurrence and require carefully monitoring during follow-up.

## Data Availability

No datasets were generated or analysed during the current study.

## Abbreviations

UAM: Unicystic ameloblastoma
OPT: Orthopantomography
CBCT: Cone beam computed tomography
WHO: World Health Organization
